# When Vomiting Becomes Neurological: A Case of Wernicke’s Encephalopathy Secondary to Severe Hyperemesis Gravidarum in the Second Trimester

**DOI:** 10.7759/cureus.119188

**Published:** 2026-11-05

**Authors:** Sam Ryan, Lauren Best, Llewelyn Jones, Lauren Donnelly

**Affiliations:** 1 Internal Medicine, Aneurin Bevan University Health Board, Cwmbran, GBR; 2 Radiology, Swansea Bay University Health Board, Swansea, GBR; 3 Geriatric Medicine, Aneurin Bevan University Health Board, Cwmbran, GBR

**Keywords:** hyperemesis gravidarum (hg), missed miscarriage, refeeding syndrome (rs), thiamine or vitamin b1 deficiency, wernicke’s encephalopathy (we)

## Abstract

Wernicke’s encephalopathy is an acute neurological emergency caused by thiamine deficiency and is an underrecognized complication of hyperemesis gravidarum. Prolonged vomiting during pregnancy can lead to severe nutritional deficiencies, increasing the risk of developing this potentially life-threatening condition. We report the case of a 35-year-old woman who experienced severe hyperemesis gravidarum, resulting in three hospital admissions and approximately 19 kg of weight loss. At 15 weeks’ gestation, following the diagnosis of a missed miscarriage, she developed blurred vision, ptosis, dizziness, and an unsteady gait. Examination revealed bilateral horizontal nystagmus and impaired right eye abduction, without confusion. Based on these clinical findings and characteristic MRI abnormalities involving the medial thalami and mammillary bodies, a diagnosis of Wernicke’s encephalopathy was made, with some biochemical features suggestive of refeeding syndrome. Treatment with intravenous Pabrinex, followed by oral thiamine and dietary support, resulted in complete neurological recovery and resolution of the imaging abnormalities. This case highlights the importance of early recognition and prompt parenteral thiamine replacement in patients with prolonged vomiting and neurological symptoms, even without the complete classical triad.

## Introduction

Wernicke’s encephalopathy is an acute neurological disorder classically presenting with a triad of ophthalmoplegia, ataxia, and confusion. It results from a deficiency of vitamin B1 (thiamine), which is an essential cofactor in cellular energy metabolism, particularly carbohydrate metabolism [1]. Although traditionally associated with alcohol dependence, Wernicke’s encephalopathy can also occur in other conditions associated with malnutrition, including pregnancy, where it is an important but underrecognized complication of severe hyperemesis gravidarum [2,3].

Pregnancy represents a particularly vulnerable state for thiamine deficiency due to increased maternal metabolic demands and the additional nutritional requirements of the developing fetus and placenta [4]. Thiamine is a water-soluble vitamin with limited bodily reserves, meaning that adequate dietary intake is required to maintain sufficient levels. Prolonged nausea and vomiting, as seen in hyperemesis gravidarum, can substantially reduce nutritional intake and impair thiamine absorption [5]. Consequently, thiamine stores can become rapidly depleted, potentially resulting in neurological complications, including Wernicke’s encephalopathy and peripheral neuropathy [2-5]. The risk is particularly significant in women experiencing persistent vomiting over several weeks, with neurological manifestations frequently developing during the first or second trimester [2,3].

Thiamine plays a crucial role in normal neurological function, acting as an essential cofactor for several enzymes involved in cerebral energy metabolism, including pyruvate dehydrogenase, α-ketoglutarate dehydrogenase, and transketolase. Given the brain’s high metabolic demands and reliance on glucose for energy, thiamine deficiency can rapidly impair neuronal energy production, leading to cellular dysfunction and neurological injury. This can particularly affect vulnerable brain regions, such as the medial thalami and mammillary bodies, which are commonly involved in Wernicke’s encephalopathy [1,6]. If left untreated, the condition can result in permanent neurological damage, including persistent cognitive impairment and Korsakoff syndrome. Early recognition and prompt treatment with parenteral thiamine are therefore essential to prevent progression and maximize the likelihood of neurological recovery. Diagnosis can be challenging, as the classical triad of ophthalmoplegia, ataxia, and confusion is often incomplete; therefore, treatment should not be delayed while awaiting confirmatory imaging or laboratory investigations [1,6].

We present a case of Wernicke’s encephalopathy in a patient following a miscarriage after a pregnancy complicated by severe hyperemesis gravidarum.

## Case presentation

A 35-year-old woman was admitted at 15 weeks’ gestation for medical management of a missed miscarriage. Her obstetric history included two previous uncomplicated pregnancies.

Before this admission, her pregnancy had been complicated by hyperemesis gravidarum. She presented at eight weeks’ gestation with severe nausea and vomiting, which resulted in three hospital admissions for intravenous fluid and antiemetic therapy. During this time, she lost approximately 19 kg. No biochemical abnormalities were identified during those admissions. Her symptoms of hyperemesis gravidarum improved after 10 weeks’ gestation. However, an ultrasound at 15 weeks demonstrated no fetal heartbeat, and she was admitted for medical management of a missed miscarriage.

The patient became unwell during this admission. Although she was no longer experiencing nausea and vomiting, she developed pyrexia and tachycardia, with a maximum recorded temperature of 39.4°C. She was subsequently treated by the obstetric team for suspected septic miscarriage with intravenous Tazocin. Laboratory investigations revealed hypokalemia, with a serum potassium level of 2.7 mmol/L. She received intravenous potassium chloride and Hartmann’s solution; however, no intravenous dextrose was administered.

The patient received a total of 360 mmol of intravenous potassium chloride and 216 mmol of oral potassium chloride as 18 sachets of oral Sando-K over three days. Despite this, she remained hypokalemic (Table 1). She was also noted to have hypomagnesemia and received intravenous magnesium replacement. Her electrocardiogram at this time was normal.

**Table 1:** Serial serum electrolyte measurements before and during hospital admission Serum potassium, magnesium, and phosphate levels were measured before and during hospital admission, demonstrating hypokalemia and persistent hypomagnesemia despite initial electrolyte replacement. N/A: not available

Electrolyte	10 weeks’ gestation	Admission (15 weeks' gestation)	Day 1 post admission	Day 2 post admission	Day 3 post admission	Day 4 post admission	Day 5 post admission	Day 6 post admission	Day 7 post admission	Day 8 post admission	Day 9 post admission	Day 10 post admission	Day 18 post admission
Potassium (mmol/L)	3.5	2.7	2.9	2.8	2.7	2.7	2.9	2.9	3.4	3.5	3.4	3.6	3.9
Magnesium (mmol/L)	N/A	N/A	0.72	N/A	0.59	0.68	0.78	0.6	0.56	0.56	0.56	N/A	0.78
Phosphate (mmol/L)	0.93	0.93	N/A	N/A	N/A	0.99	N/A	1.02	1.38	1.2	1.16	N/A	1.07

A medical consultation was requested for hypokalemia and hypomagnesemia. During the assessment, the patient reported a three-day history of blurred vision that improved when she closed one eye. She also noticed eyelid drooping, presyncope, and dizziness.

On examination, she had an unsteady, broad-based gait and variable ptosis without fatigability. The most significant findings were bilateral coarse horizontal nystagmus and an inability to abduct the right eye. No other cerebellar signs were present, and examination of the upper and lower limbs was otherwise normal.

She subsequently underwent brain CT and MRI. MRI revealed signal abnormalities in the medial thalami and mammillary bodies, consistent with Wernicke’s encephalopathy (Figure 1).

**Figure 1:**
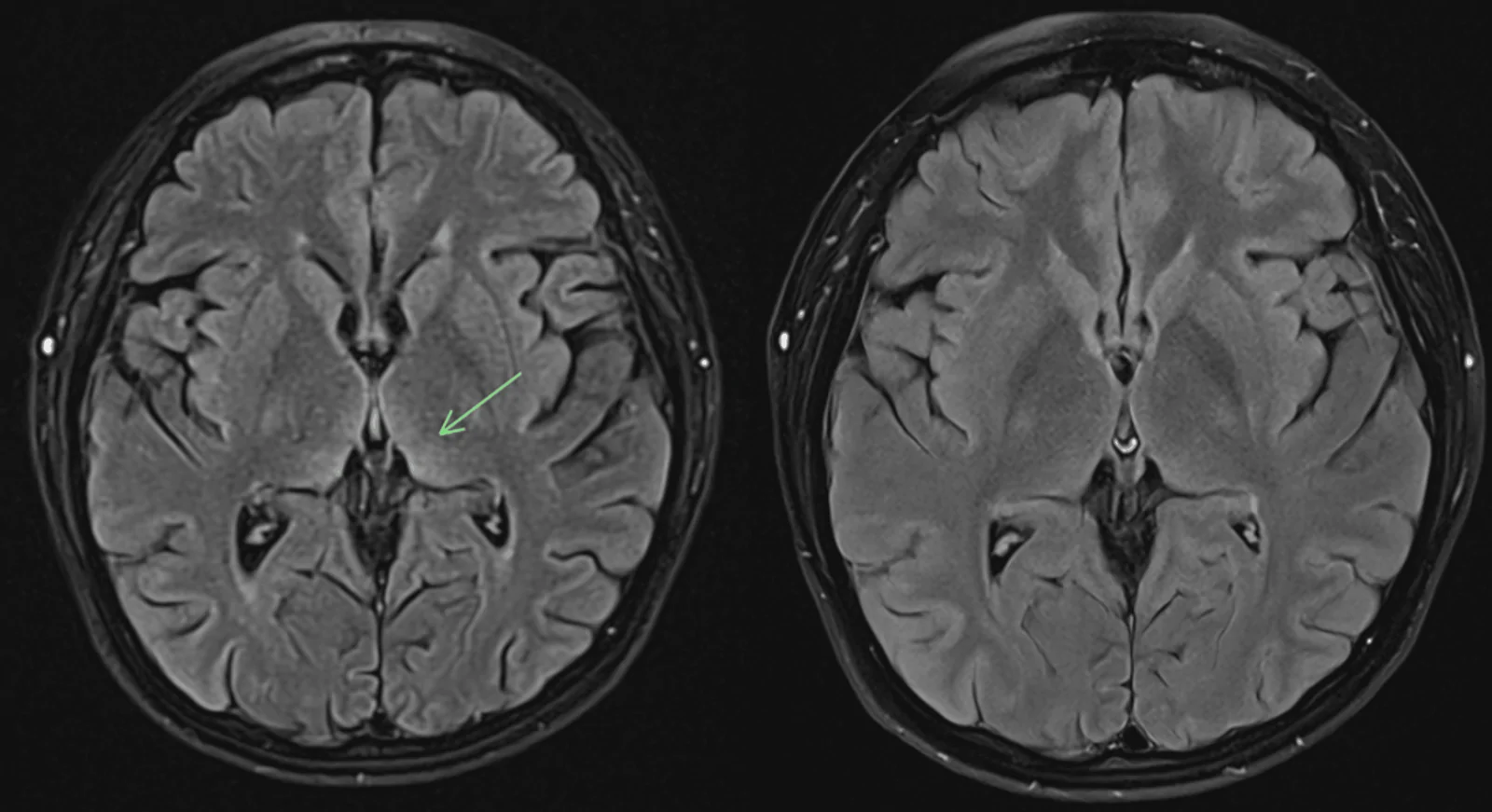
MRI brain T2 FLAIR sequence The left image shows characteristic hyperintensity in the medial thalami associated with Wernicke’s encephalopathy, which is indicated by the arrow. The right image demonstrates complete resolution of the abnormality on follow-up imaging three months later. MRI: magnetic resonance imaging, FLAIR: fluid-attenuated inversion recovery

She was subsequently diagnosed with hyperemesis gravidarum-induced Wernicke’s encephalopathy, with features suggestive of refeeding syndrome. Refeeding syndrome was suspected because electrolyte disturbances developed after her prolonged hyperemesis gravidarum symptoms had resolved. In particular, she developed hypokalemia and persistent hypomagnesemia, requiring electrolyte replacement. The temporal association between resolution of prolonged vomiting and the subsequent electrolyte derangements raised the possibility of refeeding-related metabolic disturbances following a period of significant nutritional deprivation.

She received high-dose parenteral thiamine as an intravenous vitamin B and C compound injection (Pabrinex®) for five days, followed by oral thiamine at a dose of 100 mg twice daily for one month. She was also evaluated by a dietitian. Following clinical improvement, she was discharged with plans for outpatient neurology follow-up and a repeat brain MRI after three months.

Follow-up imaging showed complete resolution of the previously reported abnormalities. The patient’s signs and symptoms also resolved completely.

## Discussion

Wernicke’s encephalopathy is an acute neurological emergency arising from thiamine deficiency. Although most cases are associated with alcohol dependence [1], Wernicke’s encephalopathy can also occur in other conditions associated with malnutrition. Pregnancy represents a particularly vulnerable state because of increased nutritional demands [1,3]. Thiamine plays an essential role in glucose metabolism, and its deficiency significantly impairs this process in neuronal cells, ultimately resulting in the neurological complications associated with Wernicke’s encephalopathy [1,6]. Thiamine replacement often causes complete resolution of the disorder [7].

Hyperemesis gravidarum is a common complication of pregnancy characterized by severe nausea and vomiting, typically presenting in the fourth to eighth week of pregnancy and resolving in the second trimester. Initial treatment typically involves dietary modification and antiemetics. However, severe cases may result in malnutrition and require hospitalization for intravenous antiemetics and fluid replacement [8].

Wernicke’s encephalopathy is a recognized but underreported complication of hyperemesis gravidarum. The risk of developing Wernicke’s encephalopathy increases if intravenous dextrose is administered before thiamine replacement. The Royal College of Obstetricians and Gynaecologists recommends thiamine supplementation in women admitted with hyperemesis gravidarum, particularly before dextrose administration [8]. A systematic review of 82 reported cases associated with hyperemesis gravidarum identified a mean maternal age of approximately 26 years, with neurological manifestations typically developing during the second trimester following several weeks of vomiting [2]. A further systematic review highlighted the serious consequences of thiamine deficiency in pregnancy and the importance of early recognition and treatment [3]. Other authors have also reported cases of Wernicke’s encephalopathy associated with hyperemesis gravidarum, further emphasizing the importance of recognizing neurological complications in pregnant patients with prolonged vomiting [9].

Our case demonstrates why Wernicke’s encephalopathy should be suspected in patients presenting with prolonged vomiting and neurological symptoms. The classical triad of ophthalmoplegia, ataxia, and confusion is often incomplete, as it was in this case, and this can lead to underrecognition of the condition. Diagnostic criteria [10] highlight the importance of considering dietary deficiency, ocular abnormalities, and cerebellar dysfunction, even without the complete classical triad. Although these criteria were developed in patients with chronic alcohol use, they reinforce the importance of clinical assessment in suspected Wernicke’s encephalopathy. Additionally, magnesium deficiency may contribute to Wernicke’s encephalopathy, as magnesium is an essential cofactor for thiamine-dependent enzymatic reactions. Hypomagnesemia can therefore impair thiamine utilization, resulting in a functional thiamine deficiency. Our patient had documented hypomagnesemia during admission, which may have further contributed to her susceptibility to Wernicke’s encephalopathy [11]. Given the significant potential complications, prompt treatment with thiamine supplementation should be provided to any patient at risk [6,7].

Our patient presented without confusion, and additional neurological signs were identified on examination. This illustrates a diagnostic challenge in surgical specialties, where neurological examinations may not be routinely performed, potentially delaying the diagnosis. MRI can provide supportive evidence, typically demonstrating bilateral, symmetric hyperintensities on T2-weighted and fluid-attenuated inversion recovery sequences. These abnormalities most commonly involve the medial thalami, mammillary bodies, periaqueductal gray matter, and tectal plate, reflecting the vulnerability of these metabolically active regions to thiamine deficiency [12]. Additional atypical sites of involvement can include the cerebellum, cranial nerve nuclei, basal ganglia, and cerebral cortex. In our patient, MRI demonstrated characteristic signal abnormalities involving the medial thalami and mammillary bodies, supporting the diagnosis of Wernicke’s encephalopathy. Subsequent follow-up imaging demonstrated complete resolution of these abnormalities.

However, this case also demonstrates that prompt parenteral thiamine administration following recognition of Wernicke’s encephalopathy can lead to substantial or even complete neurological recovery [7]. It also illustrates how multidisciplinary management involving obstetricians, physicians, dietitians, and other allied health professionals can help identify at-risk patients and prevent neurological sequelae. Our patient’s presentation also had features suggestive of refeeding syndrome, highlighting the importance of recognizing nutritional complications following prolonged vomiting and significant weight loss. Appropriate thiamine supplementation, electrolyte monitoring, and cautious nutritional rehabilitation are important in patients at risk of refeeding complications [13].

## Conclusions

Wernicke’s encephalopathy is a potentially life-threatening but treatable neurological complication of pregnancy, most commonly associated with prolonged vomiting and thiamine deficiency in the setting of hyperemesis gravidarum. Its nonspecific clinical presentation and the frequent absence of the classical triad may contribute to delayed diagnosis and adverse maternal and fetal outcomes. A high index of clinical suspicion, prompt parenteral thiamine administration, and appropriate nutritional support are essential to prevent irreversible neurological sequelae. This case highlights the importance of early recognition and timely multidisciplinary management of Wernicke’s encephalopathy in pregnancy.
